# Drug-Resistant Characteristics, Genetic Diversity, and Transmission Dynamics of Rifampicin-Resistant Mycobacterium tuberculosis in Hunan, China, Revealed by Whole-Genome Sequencing

**DOI:** 10.1128/spectrum.01543-21

**Published:** 2022-02-16

**Authors:** Wencong He, Yunhong Tan, Chunfa Liu, Yiting Wang, Ping He, Zexuan Song, Dongxin Liu, Huiwen Zheng, Aijing Ma, Bing Zhao, Xichao Ou, Hui Xia, Shengfen Wang, Yanlin Zhao

**Affiliations:** a National Institute for Communicable Disease Control and Prevention, Chinese Center for Disease Control and Prevention, Beijing, China; b Hunan Provincial Chest hospital, Tuberculosis Control Institution of Hunan province, Changsha, Hunan, China; c National Tuberculosis Reference Laboratory, Chinese Center for Disease Control and Prevention, Beijing, China; d Shenzhen Third People’s Hospital, Shenzhen City, Guangdong, China; e Beijing Pediatric Research Institute, Beijing Children’s Hospital, Capital Medical University, National Center for Children’s Health, Beijing, China; Johns Hopkins University School of Medicine

**Keywords:** *Mycobacterium tuberculosis*, rifampicin resistance, genetic diversity, whole-genome sequencing, transmission dynamics

## Abstract

To gain a deep insight into the additional drug-resistant profiles, genetic diversity, and transmission dynamics of rifampicin-resistant tuberculosis (RR-TB) circulating in Hunan province, drug susceptibility testing and whole-genome-sequencing were performed among RR-TB strains collected from Jan. 2013 to Jun. 2018 in Hunan province. A total of 124 RR-TB strains were recovered successfully and included into the final analysis. Lineage 2.2.1 was the dominant sublineage, accounting for 72.6% (90/124), followed by lineage 4.5 (11.3%, 14/124), lineage 4.4 (8.1%, 10/124), lineage 4.2 (6.5%, 8/124) and lineage 2.2.2 (1.6%, 2/124). Overall, 83.1% (103/124) and 3.2% (4/124) of RR-TB were MDR-TB and XDR-TB, respectively. Nearly 30% of RR-TB isolates were resistant to fluoroquinolones, and 26.6% (33/124) were pre-XDR-TB. Moreover, 30.6% (38/124) of RR-TB strains were identified as phenotypically resistance to pyrazinamide. Totally, 17 clusters containing 48 (38.7%, 48/124) RR-TB strains were identified, ranging in size from 2 to 10 isolates. No significant difference was detected in clustering rate between lineage 2 and lineage 4 (*χ^2^* = 0.027, *P* = 0.870). Our study revealed the complexity of RR-TB strains circulating in Hunan province with complex additional drug-resistant profile and relatively higher clustering rates. Comprehensive information based on WGS should be used to guide the design of treatment regimens and tailor public interventions.

**IMPORTANCE** Comprehensive information such as genetic background and drug-resistant profile of MTB strains could help to tailor public interventions. However, these data are limited in Hunan province, one of the provinces with high-TB burden in China. So, this study aimed to provide us with deep insight into the molecular epidemiology of RR-TB isolates circulating in Hunan province by combining phenotypic drug susceptibility testing and whole-genome sequencing. To our knowledge, this is the first study to use whole-genome sequencing data of RR-TB strains spanning more than 5 years for molecular epidemiology analysis in Hunan province, which allows us to identify genetic background information and clustered strains more accurately. Our study revealed the complexity of RR-TB strains circulating in Hunan province with complex additional drug-resistant profile and relatively higher clustering rates. Comprehensive information based on WGS should be used to guide the design of treatment regimens and tailor public interventions.

## INTRODUCTION

Tuberculosis (TB) was the top killer of human death from a single infectious organism before COVID-19 pandemic, causing approximately 10 million new cases and 1.2 million deaths annually (1). The emergence and wide-spread of drug-resistant tuberculosis (DR-TB), especially multidrug resistant TB (MDR-TB) and extensively drug-resistant TB (XDR-TB), has undoubtedly become a major stumbling block of TB elimination worldwide (1). According to WHO, an estimated 465,000 new rifampicin-resistant TB (RR-TB) occurred globally in 2019, of which 78% were multidrug resistant tuberculosis (MDR-TB) (1). Treatment of RR/MDR-TB is extremely challenging due to the limited treatment options, longer treatment duration, lower cure rates, and the need for more second-line anti-TB drugs with greater side effects and higher prices (2).

Since the full-genome sequence of Mycobacterium tuberculosis H37Rv was completed and published in 1998 (3), whole-genome sequencing (WGS) has been widely used in research, clinical, and routine surveillance work, including predicting drug resistance, investigating transmission chains, identifying mixed infection, and revealing evolutionary laws of Mycobacterium tuberculosis complex (MTBC) (4–8). These expanding applications provide clear insights into the molecular epidemiology of M. tuberculosis, which contribute significantly to the precise control and prevention of TB (9).

Hunan province locates in south-central China, with a total population of 73.20 million. As one of the provinces with high-TB-burden in China, Hunan has an estimated annual TB incidence rate of 94 cases per 100,000 population, higher than the national average level (66 cases per 100,000 population) (10). In addition, nearly 11% of all incident TB cases and 30% of previously treated TB cases in Hunan province were MDR-TB, respectively (11). Previous studies have shown that only 57% of TB patients with MDR-TB in Hunan province achieved successful treatment outcomes (12), suggesting that further research on MDR-TB in this region is urgently needed.

Molecular epidemiological data with high-resolution on RR-TB strains are limited in Hunan province. Therefore, this study aims to better understand the genetic diversity, drug-resistant profile and transmission dynamics of RR-TB isolates circulating in this region through whole-genome sequencing and provide scientific basis for DR-TB control and prevention.

## RESULTS

### Demographic and clinical characteristics.

Out of 134 rifampicin-resistant TB (RR-TB) strains isolated from Hunan province between 2013 and 2018, 130 strains were recovered successfully, while 6 isolates were excluded due to the failure of drug susceptibility testing or whole-genome sequencing. Thus, 124 RR-TB strains were included into the final analysis. Among the 124 isolates, 90 (72.6%) were from male patients and 34 (27.4%) were from female patients. The age of cases ranged from 15 to 81 years (mean ± standard deviation [SD], 49.1 ± 15.9). Overall, 11 (8.9%) patients had diabetes, and 7 (5.7%) had hepatitis B. Information on HIV status of the patients was not collected. The majority of TB cases (71.8%, 89/124) were newly diagnosed, while 35 (28.2%) cases had received previous treatment. Detailed demographic information and clinical characteristics of the study population are shown in Table 1.

**TABLE 1:** Socio-demographic characteristics of patients with RR-TB

Variables	Count (N = 124)	Percentage (%)
Sex		
Male	90	72.6
Female	34	27.4
Age(yrs)		
<30	18	14.5
30–44	28	22.6
45–59	40	32.3
≥60	38	30.6
Residence		
Rural	87	70.2
Urban	37	29.8
Educational status		
Unable to read and write	14	11.3
Primary/Middle school	95	76.6
High school and above	15	12.1
Occupation		
Farmer	84	67.7
Others	40	32.3
Diabetes (self-report)		
Yes	11	8.9
No	113	91.1
Hepatitis B (self-report)		
Yes	7	5.7
No	117	94.3
Previous TB treatment		
Yes	35	28.2
No	89	71.8

### Phenotypic drug-resistant profile.

Out of 124 rifampicin-resistant TB (RR-TB) strains, 103 (83.1%) isolates had additional resistance to isoniazid, whereas 30.6% (38/124), 33.9% (42/124), and 48.4% (60/124) were resistant to pyrazinamide, ethambutol, and streptomycin, respectively. In addition, 29.8% (37/124) of RR-TB strains were detected with fluoroquinolones resistance, while 4.8% (6/124) were resistant to both amikacin and kanamycin. As for drug-resistant patterns, 103 (83.1%, 103/124) of RR-TB strains met the definition of MDR-TB, including 66 (53.2%) strains of simple MDR-TB, 33 (26.6%) of pre-XDR-TB and 4 (3.2%) of XDR-TB. Only 16 (12.9%) RR-TB strains were mono-DR-TB, and 5 (4.0%) RR-TB strains were poly-DR-TB. In addition, 35.9% (37/103), 39.8% (41/103), 56.3% (58/103), 34.0% (35/103) of the MDR-TB strains were observed with additional resistance to pyrazinamide, ethambutol, streptomycin, and fluoroquinolones, respectively. Six (6/103, 5.8%) of MDR-TB strains were resistant to both amikacin and kanamycin. Overall, 64.1% (66/103) of MDR-TB were simple MDR-TB, while 32.0% (33/103) and 3.9% (4/103) were Pre-XDR-TB and XDR-TB, respectively (Table 2).

**TABLE 2:** Drug-resistant profiles of 124 RR-TB and 103 MDR-TB strains^*a*^

Drugs/drug resistant patterns	Resistance among RR-TB (N = 124)		Resistance among MDR-TB (N = 103)	
	no.	% (95%CI)	no.	% (95%CI)
First-line drugs				
Rifampicin	124	100.0 (96.3, 100.0)	103	100.0 (95.5, 100.0)
Isoniazid	103	83.1 (75.0, 89.0)	103	100.0 (95.5, 100.0)
Pyrazinamide	38	30.6 (22.9, 39.7)	37	35.9 (26.9, 46.0)
Ethambutol	42	33.9 (25.8, 43.0)	41	39.8 (30.4, 49.9)
Streptomycin	60	48.4 (39.4, 57.5)	58	56.3 (46.2, 65.9)
Second-line drugs				
Moxifloxacin	35	28.2 (20.7, 37.1)	33	32.0 (23.4, 42.1)
Ofloxacin	37	29.8 (22.1, 38.8)	35	34.0 (25.1, 44.1)
Kanamycin	6	4.8 (2.0, 10.7)	6	5.8 (2.4, 12.8)
Amikacin	6	4.8 (2.0, 10.7)	6	5.8 (2.4, 12.8)
Mono-DR-TB	16	12.9 (7.8, 20.4)	*NA*	*NA*
Poly-DR-TB	5	4.0 (1.5, 9.6)	*NA*	*NA*
MDR-TB	103	83.1 (75.0, 89.0)	103	100.0 (95.5, 100.0)
Simple MDR-TB	66	53.2 (44.1, 62.2)	66	64.1 (54.0, 73.1)
Pre-XDR-TB	33	26.6 (19.3, 35.4)	33	32.0 (23.4, 42.1)
XDR-TB	4	3.2 (1.0, 8.6)	4	3.9 (1.3, 10.2)

a *NA*, not applicable.

### Molecular drug-resistant characteristics.

Among 124 phenotypic RR-TB strains, 118 strains had detectable mutations in the *rpoB* gene. The most prevalent drug-resistant mutations were Ser450Leu (45.2%, 56/124), followed by His445Leu (6.5%, 8/124) and His445Tyr (6.5%, 8/124). Three (2.4%, 3/124) strains harbored gene mutations out of RRDR, namely, Ile491Phe (chromosome 761277, Rv0667 c.1471A>T), Val170Phe (chromosome 760314, Rv0667 c.508G>T), and Glu761Asp (chromosome 762089, Rv0667 c.2283G>C), respectively. Double mutations were detected in 17 (13.7%, 17/124) strains, two (1.6%, 2/124) of which also carried complementary mutations in the *rpoC* gene (Table S1). Of 103 MTB strains with phenotypic isoniazid resistance, 93 strains had detectable mutations related to isoniazid resistance. *KatG* Ser315Thr (58/103, 56.3%) was the most common mutations, followed by *KatG* Ser315Asn (10/103, 9.7%) and *fabG1* c-15t (5/103, 4.9%). Eight (8/103, 7.8%) strains carried combined mutations, while no gene mutations were detected in isoniazid-susceptible strains (Table S2). Among the 38 MTB strains with phenotypically pyrazinamide-resistance, 30 (78.9%) strains had detectable mutations in the *pncA* gene, involving 25 mutant forms, and no hot spot regions were identified. Mutations in the *pncA* gene were also detected in seven pyrazinamide-susceptible strains (Table S3). Ethambutol-resistant isolates were mainly related to the mutation *embB* Met306Val (23/42, 54.8%) and Met306Ile (6/42, 14.3%). Only one strain with ethambutol-resistant phenotype had no detectable gene mutation conferring ethambutol-resistance. Notably, 21 MTB strains carried mutations or combinations of mutations in the *embB* gene and the *embA* promoter region but were phenotypically sensitive to ethambutol (Table S4). Most of the phenotypically streptomycin-resistant strains were detected with Lys43Arg and Lys88Arg mutation in *rpsL* gene, accounting for 76.7% (46/60) and 16.7% (10/60), respectively. Three (5%, 3/60) MTB strains with streptomycin-resistant phenotype carried mutation in *rrs* gene (Table S5). Additionally, almost all phenotypically fluoroquinolone-resistant MTB strains were identified with mutations in the *gyrA* or *gyrB* gene, mainly linked to the mutation of Asp94Gly (13/37, 35.1%) and Ala90Val (10/37, 27.0%) in *gyrA* gene (Table S6). Five strains were detected with the a-1401g mutation in *rrs* gene associated with kanamycin- and amikacin-resistance (Table S7).

### Phylogenetic analysis.

**FIG 1:**
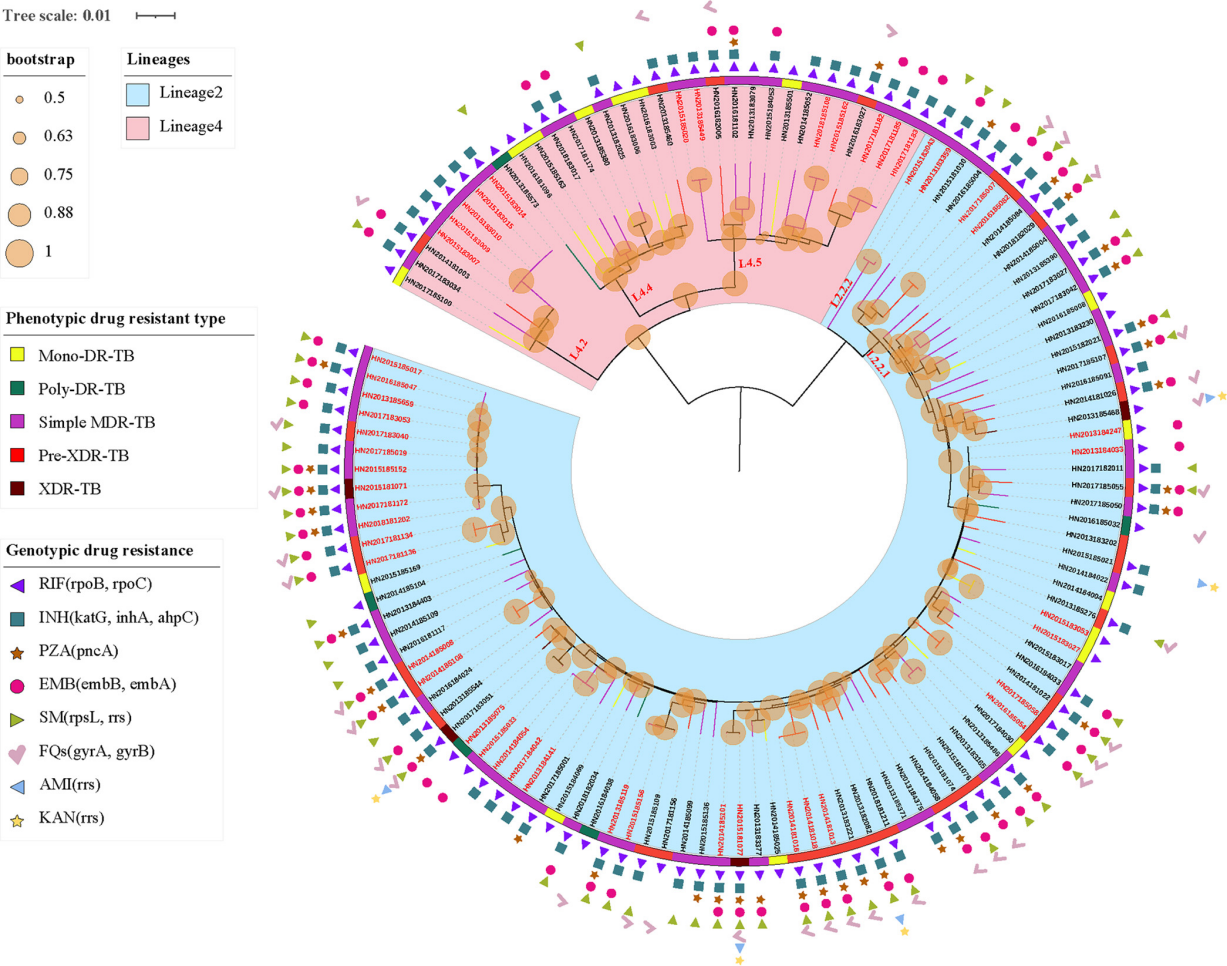
Maximum-likelihood tree of 124 rifampicin-resistant strains and annotated with drug-resistant information. Note: Lineages, bootstrap value, phenotypic drug-resistant type, and genotypic drug-resistant profile of strains are shown; The potential transmission clusters defined by no more than 12 SNPs are indicated in red on the branch tips; Branches are colored by phenotypic drug resistant type; Scale bar indicates the genetic distance proportional to the total number of single nucleotide polymorphisms.

To better understand the genetic structures and the role of recent transmission in these RR-TB strains, the phylogenetic tree was constructed based on nonredundant single-nucleotide polymorphisms (SNPs) using maximum-likelihood method (13). All RR-TB strains were phylogenetically classified according to the SNPs barcode nomenclature proposed by Coll et al. (14). Overall, two main-linages were identified: 74.2% (92/124) of RR-TB strains were assigned to lineage 2 (East Asian genotype) and 25.8% (32/124) to lineage 4 (Euro-American genotype). Lineage 2.2.1 (Beijing genotype) was the dominant sublineage, accounting for 72.6% (90/124), followed by lineage 4.5 (14/124, 11.3%), lineage 4.4 (10/124, 8.1%) and lineage 4.2 (8/124, 6.5%). Only 2 (1.6%, 2/124) isolates belong to lineage 2.2.2 (Fig. 1). Interestingly, lineage 2 was more likely to be resistant to pyrazinamide, ethambutol, streptomycin, moxifloxacin, and ofloxacin than lineage 4 (*P *< 0.05) (Table 3). Pre-XDR-TB strains were significantly more likely to be present in lineage 2 (*P* = 0.036) (Table 3).

**TABLE 3:** Comparison of drug-resistant profiles between lineage 2 and lineage 4^*a*^

Drugs/drug resistant patterns	Lineage 2 (*n* = 92)	Lineage 4 (*n* = 32)	χ^2^	*P*
	no. (%)	no. (%)		
First-line drugs				
Rifampicin	92 (100.0)	32 (100.0)	*NA*	*NA*
Isoniazid	79 (85.9)	24 (75.0)	1.994	0.158
Pyrazinamide	35 (38.0)	3 (9.4)	9.181	0.002
Ethambutol	41 (44.6)	1 (3.1)	18.203	<0.001
Streptomycin	56 (60.9)	4 (12.5)	22.242	<0.001
Second-line drugs				
Moxifloxacin	31 (33.7)	4 (12.5)	5.265	0.022
Ofloxacin	33 (35.9)	4 (12.5)	6.194	0.013
Kanamycin	6 (6.5)	0 (0.0)		0.337*
Amikacin	6 (6.5)	0 (0.0)		0.337*
Mono-DR-TB	9 (9.8)	7 (21.9)	3.089	0.079
Poly-DR-TB	4 (4.3)	1 (3.1)		>0.999*
MDR-TB	79 (85.9)	24 (75.0)	1.994	0.158
Simple MDR-TB	46 (50.0)	20 (62.5)	1.490	0.222
Pre-XDR-TB	29 (31.5)	4 (12.5)	4.399	0.036
XDR-TB	4 (4.3)	0 (0.0)		0.572*

a *NA*, not applicable; * indicates *P* value was calculated by Fisher exact test.

**FIG 2:**
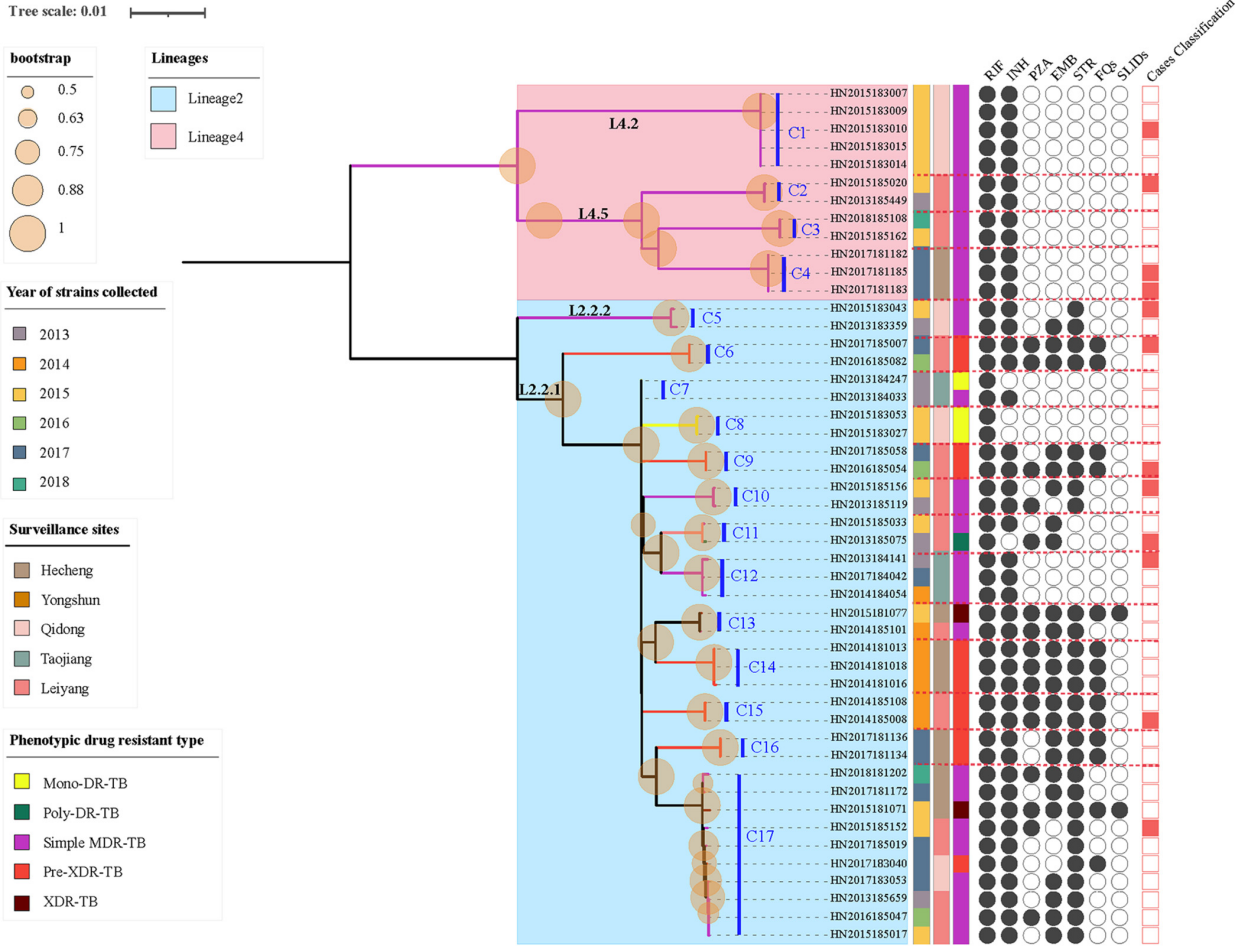
Maximum-likelihood tree of 44 rifampicin-resistant tuberculosis strains within 17 clusters and their phenotypic drug-resistant profiles. Note: The red dotted lines indicate boundaries of individual clusters; Cluster 1–17 was labeled as C1-C17; RIF, rifampicin; INH, isoniazid; PZA, pyrazinamide; EMB, ethambutol; STR, streptomycin; FQs, fluoroquinolones, including moxifloxacin and ofloxacin in this study; SLIDs, second-line anti-TB drugs, including kanamycin and amikacin in this study; Circles filled with black indicate drug-resistant strains, while empty circles indicate drug-susceptible strains; Rectangle filled with red indicate strains collected from cases with previously treatment history, while empty rectangles indicate strains collected from new cases; Scale bar indicates the genetic distance proportional to the total number of single nucleotide polymorphisms.

Genomic transmission clusters were defined using 12 SNPs as a cutoff value (15). Overall, 17 putative clusters (C1-C17) containing 48 (38.7%, 48/124) RR-TB strains were identified by integrating SNPs data and clinical information, ranging in size from 2 to 10 isolates (Fig. 2). Three-quarters (36/48, 75.0%) of the clustered strains were isolated from new cases. 10 (10/17, 58.8%) of the 17 clusters (C2, C3, C5, C6, C9, C10, C11, C12, C13, C17) contained strains spanning years, and the largest cluster (C17) involved strains spanning more than 5 years. In addition, 75.0% (36/48) of isolates within a cluster (C1, C2, C3, C4, C5, C6, C7, C8, C9, C10, C11, C12, C14, C15, C16, C17) were collected from the same surveillance sites, sharing geographical links. The clustering rate for lineage 2 was 39.1% (36/92), which was approximately equal to that of lineage 4 (37.5%, 12/32), and there was no statistical difference in the clustering rate between these two lineages (χ2 = 0.027, *P* = 0.870) (Table 4). The percentages of Mono-DR-TB, Poly-DR-TB, simple MDR-TB, Pre-XDR-TB, and XDR-TB isolates that fell into clusters were 18.8% (3/16), 20.0% (1/5), 45.5% (30/66), 36.4% (12/33), and 50.0% (2/4), respectively (Fig. 2). Seven clusters (C5, C7, C9, C10, C11, C13, C17) included isolates with different phenotypic drug-resistant spectrums, of which, 2 clusters (C7, C13) included isolates with progressively increasing drug-resistant profiles in relation to chronology, while 5 clusters (C5, C9, C10, C11, C17) included older isolates (strain with earlier year of isolation) with broader drug-resistant spectrum than younger ones (strain with more recent year of isolation).

**TABLE 4:** Comparison of clustering rate between lineage 2 and lineage 4

Lineages	Clustered cases	Unclustered cases	χ^2^	*P*
Lineage 2	36 (39.1)	56 (60.9)	0.027	0.870
Lineage 4	12 (37.5)	20 (62.5)		

## DISCUSSION

Our study gained a deep insight into the molecular epidemiology of RR-TB in Hunan, China, by integrating DST and WGS for detection of drug-resistant profiles, identification of genetic diversity, and analysis of potential clusters of RR-TB strains. As expected, lineage 2 (mainly Beijing genotype [lineage 2.2.1]) was the dominant genotype of RR-TB strains circulating in Hunan province (10). Drug resistance was previously thought to be associated with Beijing genotype (16, 17). Here, we provide evidence that Beijing genotype was more likely to be resistant to ethambutol, pyrazinamide, streptomycin, and fluoroquinolones. Conversely, a recent study from China reported that Beijing genotype is less associated with drug resistance (18). Further study is needed to clarify this issue, especially from a molecular evolutionary perspective. Some studies reported that lineage 2 has higher transmissibility than lineage 4 (19, 20). However, the present study found no association between the genotype and clustering rates of RR-TB strains. This result may be influenced by the sample source and the study time frame, as well as the different thresholds for defining genomic clusters (21).

Overall, more than 83% of RR-TB strains were MDR-TB, and 3.2% were XDR-TB. Previous meta-analysis revealed that more than 90% RR-TB were also with additional resistance to isoniazid, and RR-TB has been regarded as surrogate marker of MDR-TB (22). However, in the present study, 17% of RR-TB was still susceptible to isoniazid, suggesting isoniazid might not be excluded from treatment regimen for initial RR-TB patients. Worryingly, more than 35% of MDR-TB strains were identified as phenotypically resistant to pyrazinamide, which was similar to Shanghai (38.5%) and United States (38.0%), while higher than data obtained from Guangxi (30.8%), Heilongjiang (28.6%), and Sichuan (33.3%) (23, 24). Additionally, both phenotypic and genotypic drug resistant profile revealed a worrying situation concerning fluoroquinolones resistance. Nearly 35% of MDR-TB were detected with additional resistance to fluoroquinolones, known as pre-XDR-TB, which is only one step away from XDR-TB. Comparison of our data with the results of the national survey in 2008 (25), the resistant rate of MDR-TB against fluoroquinolones was significantly increased. This finding was supported by a recent study from Xia Hui et al. (26). Currently, pyrazinamide and fluoroquinolones remain the cornerstones for treatment of MDR/RR-TB in China until novel drugs and regimens become available on a large scale (27, 28). The severe situation of drug resistance against pyrazinamide and fluoroquinolones highlights the urgent need for resistance detection before designing treatment regimen.

Concerning the molecular mechanism of resistance to anti-TB drugs, gene mutations were screened based on whole-genome data by using a reliable online tool, TB profiler (29). In concordance with previous studies, analysis of rifampicin and isoniazid resistant mutations showed a clear predominance of well-established mutations in the rifampicin resistance-determining region (RRDR) and *katG* 315 codon (30–33). The detection of gene mutations out of RRDR in RR-TB strains is not a rare phenomenon, as Siu reported previously, highlighting the need to promote whole-genome sequencing for rapid drug resistance prediction (34). Moreover, whole-genome sequencing has been included in WHO’s guidelines for drug-resistance prediction because it overcomes many of the significant challenges associated with conventional phenotypic testing as well as limitations of less other molecular tests (35). Our study demonstrated that mutations in *pncA* gene confer 78.9% of pyrazinamide-resistance, consistent with Xia’s study conducted in Zhejiang, China (36). Several studies have shown that the prevalence of *pncA* mutation among pyrazinamide-resistant strains varies considerately across regions, ranging from 45.7% in Brazil (37), 70.6% in Iran (38), 75.0% in Thailand (39), and 94.1% in Sweden (40). However, in line with many studies (28, 41), a high diversity of *pncA* mutation associated with pyrazinamide resistance was found in our study, which support the idea that purification selection against *pncA* is relatively weak (42). This diversity of *pncA* gene mutation makes the development of a molecular test for rapid identification of pyrazinamide-resistance difficult (43). More importantly, in our study, some TB strains with phenotypically resistance against anti-TB drugs (e.g., rifampicin, isoniazid, pyrazinamide, fluoroquinolones, etc.) had no detectable gene mutations, suggesting that alternative mechanisms, such as drug efflux pump and decreased cell wall permeability to drugs, may also be related to drug resistance in MTBC (44–46).

Besides the drug-resistant profiles, we also investigated the transmission dynamics of RR-TB strains. The clustering rate of up to 30% indicates that recent transmission does play an important role in the incidence of RR-TB in Hunan province. In China, most primary care facilities, especially in remote rural areas, only perform sputum cultures on specimens from sputum smear-positive patients. Based on this situation, TB patients with sputum smear-negative but culture-positive may be delayed in treatment due to missed diagnoses, which can lead to a longer period of infectiousness and ongoing transmission of TB (21). Currently, there is no consensus on the relationship between drug resistance and the transmissibility of MTB strains. Some researchers believed that the fitness cost caused by drug-resistant mutation could reduce the chance of transmission (47). Some studies suggested that delayed initiation of adequate and effective therapy can prolong the infectious period of patients with DR-TB, thus causing further transmission events (48). The comparative analysis of drug-resistant patterns and clustering rate of RR-TB strains in this study seems to support the latter view.

Our study found 75.0% of isolates within a cluster were collected from the same county, indicating that the recent transmission mainly occurred in local area. In some clusters, isolates exhibited different drug-resistant types in relation to chronology, suggesting the progression and accumulation of mutations linked to drug resistance (19). Interestingly, some clusters included isolates of different drug-resistant types and ancestral isolates carried more drug-resistant mutations than their descendants. This result could be partially explained by the different durations of latent phase after transmission events, resulting in cases infected with more troublesome isolates (e.g., XDR-TB) emerging earlier than those with less troublesome strains (e.g., MDR-TB) (49). Another possible explanation is that not all cases from the potential cluster were enrolled in this study, and some index cases with primary resistance may have been missed in the selected population (19).

One major strength of this study is that we used whole-genome sequencing data from RR-TB strains spanned over 5 years to perform cluster analysis based on SNPs differences, which enable us to identify clustered strains more accurately. There are several limitations in our study. First, the proportion of recent transmission events might be underestimated since only RR-TB cases with sputum smear-positive were included in this study. Second, RR-TB strains we analyzed were only from five drug resistance surveillance sites in Huan province. Thus, the generalizability of the results obtained in this study may be limited. Third, due to the retrospective nature of this study, treatment outcome and HIV status of TB patients were not collected during the national drug-resistance surveillance, which prevented us from exploring the correlation between drug-resistance profile and clinical outcomes. Lastly, limited by the small sample size, we did not analyze the correlation between the sublineage and drug resistant characteristics of MTB strains.

In conclusion, lineage 2.2.1 dominates the prevalence of RR-TB in Hunan province, and our efforts to mitigate the challenge of RR-TB should be focused on this genotype. The severe situation of drug resistance against pyrazinamide and fluoroquinolones warns us to pay attention to the rational use of these two drugs. In addition, given the important role of recent transmission in the incidence of RR-TB, targeted interventions such as TB cases management and active case finding are urgently needed to prevent further transmission of RR-TB in Hunan province. WGS, as the most promising tool for predicting drug resistance and identifying clustered strains should be used to guide the design of treatment regimens and tailor public interventions.

## MATERIALS AND METHODS

### Sample collection.

**FIG 3:**
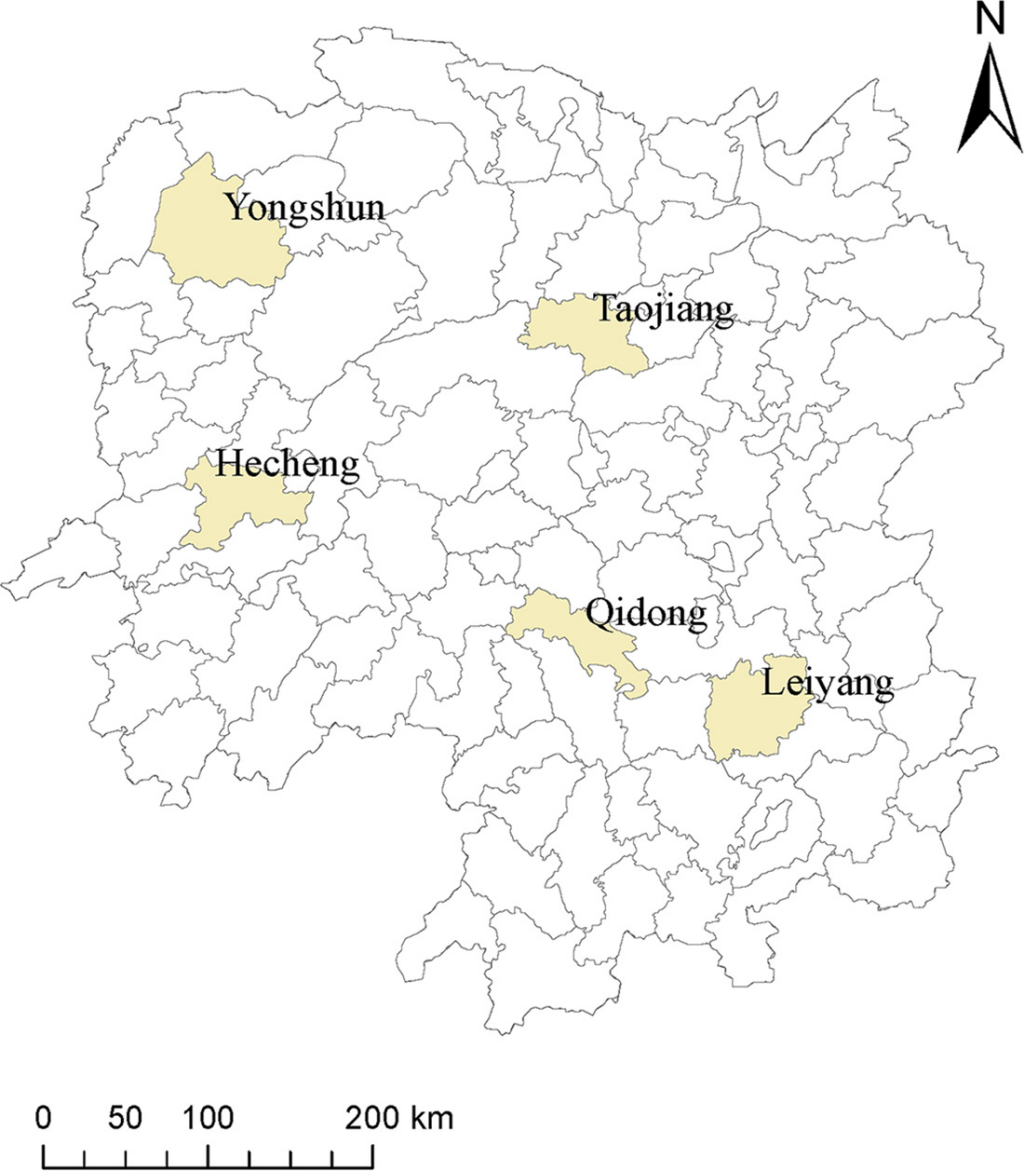
Distribution of five drug resistance surveillance sites in Hunan province.

This was a retrospective study based on routine national drug resistance surveillance work in Hunan province. The study sample comprised all rifampicin-resistant MTB strains isolated from suspected pulmonary tuberculosis patients with sputum smear-positive who visited local designated hospitals or dispensaries in the five surveillance sites (Hecheng, Yongshun, Qidong, Taojiang, Leiyang) in Hunan province between January 2013 and Juan 2018. The surveillance sites (Fig. 3) selection referred to the first national survey of drug-resistance (25). All rifampicin-resistant MTB isolates were previously identified using proportion method on Lowenstein-Jensen medium containing rifampicin at a concentration of 40 μg/mL (21) and then stored at −80°C. A total of 134 rifampicin-resistant TB strains isolated from unique TB patients were obtained through the routine national drug resistance surveillance work in Hunan province between January 2013 and June 2018. These strains were thawed and subcultured on the Löwenstein-Jensen (L-J) medium for further analysis by combining phenotypic drug susceptibility testing (DST) and whole-genome sequencing (WGS). Serial samples from the identical patients were excluded from this study.

### Patient information.

Demographic information (gender, age, address, occupation, and educational status) and clinical characteristics (complications and previous TB treatment history) of patients were extracted from the national drug resistance surveillance database, which was collected and compiled by local medical staff using questionnaires at the time of patient visits.

### Drug susceptibility testing (DST).

Drug susceptibility of MTB strains against rifampicin, isoniazid, ethambutol, streptomycin, kanamycin, amikacin, moxifloxacin and ofloxacin was determined using MYCOTB plate (Thermo Fisher Scientific Inc., USA) in this study, which has been reported as an alternative DST method with high accuracy and reproducibility (50). All steps were conducted strictly according to the manufacturer’s instructions by trained staff at the national tuberculosis reference laboratory of China. Briefly, 0.5 McFarland suspension of M. tuberculosis was prepared by Ultrasonic Milling Instrument (TB Healthcare, China) from fresh colonies grown on L-J medium. Suspensions were diluted 100-fold with the addition of 100 μL of the 0.5 McFarland suspension to 10 mL of Middlebrook 7H9 Broth with 10% Oleic Albumin Dextrose Catalase (OADC). Aliquots of 100 μL of standard 1.5 × 10^5^ CFU/mL inoculum were distributed to each well by the semiautomated Sensititre Auto-inoculator (Thermo Fisher, Scientific Inc., USA). All plates were sealed with the adhesive membranes and incubated at 37°C in 5% CO_2_. After incubation for 10–21 days, the DST results were read separately by two trained operators using the Vizion Digital viewing System (51, 52). Inconsistent results need to be reread by a third experienced experimenter. MIC was defined as the lowest antibiotic concentration that will inhibit the visible growth of a microorganism. All results are valid unless there are no skipped wells (bacterial growth is observed in wells containing higher concentrations of anti-TB drug, while not in wells containing lower concentration of anti-TB drug), no contamination, and both positive-control wells have distinct bacterial growth. H37Rv (ATCC 27294) was used as pan-susceptible control in each batch of drug susceptibility testing. The concentration range and the breakpoint concentration of each drug included in this study are shown in Table 5. All DSTs for each isolate were performed in duplicate.

**TABLE 5:** The concentration range and critical concentration of anti-TB drugs included in MYCOTB plate

Drug	Concentration range (μg/mL)	Critical concentration (μg/mL)
Rifampicin	0.12–16	1
Isoniazid	0.03–4	0.2
Ethambutol	0.5–32	5
Streptomycin	0.25–32	2
Moxifloxacin	0.06–8	0.5
Ofloxacin	0.25–32	2
Kanamycin	0.6–40	5
Amikacin	0.12–16	5

Pyrazinamide susceptibility testing was performed using Bactec MGIT 960 liquid culture system (Becton, Dickinson Diagnostic System, NJ, USA). The Bactec MGIT 960 PZA kit allows susceptibility testing in modified Middlebrook 7H9 broth (PH 5.9) at a pyrazinamide concentration of 100 μg/mL. All procedures were carried out strictly according to the instructions described previously (53, 54). H37Rv (ATCC 27294) and Mycobacterium bovis BCG (ATCC 34540) were used as susceptible and resistant controls, respectively.

### DNA extraction and sequencing.

All rifampicin-resistant MTB strains were scraped from L-J slant, and genomic DNA were extracted using the cetyltrimethylammonium bromide (CTAB) method as previously described (55). Sequencing libraries were prepared by using the Illumina Nextera kit following the manufacturer’s protocol and sequenced on Illumina Hiseq X 10 (Illumina, Inc.) with 2 × 150 paired-end (PE) strategies. All whole-genome sequencing procedures were performed by Annoroad Gene Technology company (Beijing, China).

### Phylogenetic analysis.

The overall quality of sequence reads was checked using FastQC (v0.11.8) (56). Verified paired-end reads were filtered with Trimmomatic (v 0.38) using default values and a minimum Phred Quality score of 20 (57). Only filtered paired-end reads were kept for downstream analysis. Sequencing reads were mapped to the reference genome H37Rv (NC_000962.3) using BWA-MEM (v0.7.17) (58). SAMtools (v1.3.1) and GATK (v3.8.0) was used to call variants, including single nucleotide polymorphisms (SNPs) and insertion/deletions (indel) (19). The variants filtration was performed based on the following criteria: minimum coverage depth of 10 ×, Q20 minimum quality score for each variant and more than 75% allele frequency.

SNP positions in at least 95% of the isolates were concatenated to a sequence alignment, excluding SNPs that located in repetitive regions of the genome like PE/PPE-PGRS family genes, insertions, mobile elements or phage sequence. The maximum likelihood trees were constructed using MEGA-X (v.10.1.8) based on the SNP alignment parameters (59). General time reversible model was used, and the bootstraps were performed with 1,000 replicates (19). Phylogenetic tree was visualized and modified with iTOL (v 6.4.3) (https://itol.embl.de/) (60). Clusters were defined as the isolates with pairwise genetic distance less than 12 SNPs according to the previous study (15). The cluster size was defined as the number of MTB strains that are included within the identical cluster.

### Lineage and antimicrobial resistance prediction.

WGS-based drug-susceptibility prediction was performed using an TB Profiler (v3.0.8) (https://tbdr.lshtm.ac.uk/), which can detect known resistance associated polymorphisms (61). The data were screened for mutations associated with resistance to anti-TB drugs based on a curated database (Supplementary file 1). Lineage and sublineage calls of each isolate were made and verified using the fast-lineage-caller v1.0 (https://github.com/farhat-lab/fast-lineage-caller) (58).

### Statistical analysis.

Chi-square test or Fisher exact test was used for categorical data. All statistical analysis was performed in the SPSS version 18.0 software (SPSS Inc., Chicago, Illinois.). *P* < 0.05 was considered statistically significant.

### Definitions.

Mono-DR-TB was defined as MTB strain that was confirmed by *in vitro* drug susceptibility testing to be resistant to only one anti-TB drugs (rifampicin, isoniazid, pyrazinamide, ethambutol, streptomycin, moxifloxacin, ofloxacin, kanamycin and amikacin) tested in this study. Poly-DR-TB was defined as MTB resistance to at least two or more anti-TB drugs (rifampicin, isoniazid, pyrazinamide, ethambutol, streptomycin, moxifloxacin, ofloxacin, kanamycin and amikacin) but not include the concurrent resistance to rifampicin and isoniazid. MDR-TB was defined as MTB resistance to at least isoniazid and rifampicin. Simple MDR-TB was defined as an MDR-TB strain that was susceptible to both fluoroquinolones (moxifloxacin or ofloxacin) and the second-line anti-TB drugs (amikacin or kanamycin). Pre-XDR-TB was defined as MDR-TB with additional resistance to any fluoroquinolones (moxifloxacin or ofloxacin) or any second-line injectable drugs (amikacin or kanamycin), but not both. XDR-TB was defined as MDR-TB with additional resistance to any fluoroquinolones and at least 1 s-line injectable drugs.

### Ethics Statement.

National drug-resistant surveillance (DRS) was ethically approved by the Ethics Committee of Chinese Center for Disease Control and Prevention since the first national survey in 2007 (25). Ethics approval of the present study was skipped because all isolates used in this study were from previous drug-resistance surveillance (DRS) work, and demographic characteristics were extracted from previous data sets and no additional data and specimens were collected. Each patient signed an informed consent form during the routine DRS.

### Data availability.

Data of this study will be fully available and without restriction upon reasonable request.
