# Supplementary material for: Drug-Resistant Characteristics, Genetic Diversity, and Transmission Dynamics of Rifampicin-Resistant Mycobacterium tuberculosis in Hunan, China, Revealed by Whole-Genome Sequencing
Source: Microbiol Spectr. 2022 Feb 16;10(1):e01543-21. doi: 10.1128/spectrum.01543-21 (PMC8849054; doi:10.1128/spectrum.01543-21)
Supplement: SUPPLEMENTAL FILE 3 — Supplemental material. Download SPECTRUM01543-21_Supp_2_seq8.pdf, PDF file, 0.2 MB [file spectrum01543-21_supp_2_seq8.pdf]

Supplementary file2

Table S1 The distribution of gene mutations associated with rifampicin-resistance

rpoB mutation (codon position)	*rpoC* mutation (codon position)	No. of isolates with resistant phenotype
Asp435Tyr		2
Asp435Val		2
Glu761Asp		1
His445Arg		3
His445Asp		5
His445Cys		1
His445Leu		8
His445Tyr		8
Ile491Phe		1
Leu452Pro		6
Leu452Pro	Ile885Val	1
Ser441Leu		2
**Ser450Leu**		**56**
Ser450Leu	Gly332Arg	1
Ser450Phe		1
Ser450Trp		3
Val170Phe		1
Leu430Pro		1
Asp435Gly+Leu430Arg		1
His445Arg+Gln429His		1
His445Gln+Leu430Pro		1
His445Tyr+Asp435Phe		5
His445Tyr+Asp435Val		1
Leu430Pro+Asp435Gly		1
Leu443Trp+Thr444Pro		2
Ser431Gly+Leu430Pro		3
WT	WT	6
Total		124

Note: WT indicates wild type.

Table S2 The distribution of gene mutations associated with isoniazid resistance

katG mutation (codon position)	*ahpC* mutation (codon position)	*fabG1* mutation (codon position)	*inhA* mutation (codon position)	No. of isolates with resistant phenotype	No. of isolates with susceptible phenotype
	g-48a			3	0
	c-52t			1	0
Asp735Ala	c-52t			1	0
	c-54t			1	0
	c-81t			1	0
	c-81t+g-74a			1	0
		c-15t		5	0
		c-15t	Ile21Val	1	0
				1	0
Tyr155Cys	c-15t			1	0
146_147insTGCA	c-81t			1	0
1856_1857insT				1	0
Asn138Asp				1	0
Asp94Gly				2	0
Ser315Asn				10	0
**Ser315Thr**				**58**	0
Ser315Thr		c-15t		1	0
Tyr155Cys		c-15t		2	0
Val1Ala				1	0
WT	WT	WT	WT	10	21
Total				103	21

Note: WT indicates wild type.

Table S3 The distribution of gene mutations associated with pyrazinamide-resistance

*pncA*mutation (codon position)	No. of isolates with resistant phenotype	No. of isolates with susceptible phenotype
A-11G	1	0
A-11G+Thr47Pro	1	0
106_106del	0	1
116_116del	1	0
177_178insC	1	0
193_202del	1	0
281_282insT	1	0
314_315insG+Tyr41*	0	1
355_355del	3	0
391_392insG	1	0
391_392insGG	0	2
405_405del	1	0
407_408insA	1	0
417_418insG	2	0
Chromosome:g.2288682_2288719del	1	0
Chromosome:g.2288682_2288841del	0	1
Chromosome:g.2288682_2288882del	1	0
Ala146Thr	1	0
Ala46Val	1	0
Cys14Trp	1	0
Ile6Thr	1	0
Leu85Arg	2	0
Phe94Leu	1	0
Thr135Pro	1	0
Thr142Ala	1	0
Thr76Ile	1	0
Thr76Pro	1	0
Val130Ala	0	1
Val139Ala	1	0
Val155Ala	0	1
Val7Gly	2	0
WT	8	79
Total	38	86

Note: WT indicates wild type; * indicates stop codon.

Table S4 The distribution of gene mutations associated with ethambutol-resistance

embA mutation (codon position)	*embB* mutation (codon position)	No. of isolates with resistant phenotype	No. of isolates with susceptible phenotype
c-12t	Tyr319Cys	1	0
c-16g		0	1
c-16t		0	1
	Asp1024Asn	0	1
	Gln497Arg	4	1
	Gly406Ala	1	0
	Gly406Asp	1	3
	Gly406Ser	0	2
	Met306Ile	6	10
	**Met306Val**	**23**	**1**
	Met306Ile+Gln497Arg	1	0
c-12t	Met306Val+Tyr319Cys	1	0
	Tyr319Ser	3	1
WT	WT	1	61
Total		42	82

Note: WT indicates wild type

Table S5 The distribution of gene mutations associated with streptomycin-resistance

*rpsL*mutation (codon position)	*rrs* mutation (codon position)	No. of isolates with resistant phenotype	No. of isolates with susceptible phenotype
**Lys43Arg**		**46**	**0**
Lys88Arg		10	0
	a-514c	2	0
	c-517t	1	2
WT	WT	1	62
Total		60	64

Note: WT indicates wild type

Table S6 The distribution of gene mutations associated with fluoroquinolones resistance

*gyrA*mutation (codon position)	*gyrB*mutation (codon position)	No. of isolates with resistant phenotype	No. of isolates with susceptible phenotype
**Ala90Val**		**10 (10)**	0 (0)
Ala90Val	Thr500Asn	1 (1)	0 (0)
Asp89Asn		1 (1)	0 (0)
Asp94Ala		2 (2)	0 (0)
Asp94Ala+Ala90Val		1 (1)	0 (0)
Asp94Asn		2 (2)	0 (0)
**Asp94Gly**		**13 (13)**	0 (0)
Asp94Tyr		3 (3)	0 (0)
Ser91Pro		2 (2)	0 (0)
	Asp461Asn	1 (0)	0 (1)
WT	WT	1 (0)	87 (88)
Total		37 (35)	87 (89)

Note: WT indicates wild type; the numbers in parentheses represent the resistance to moxifloxacin, while the numbers outside the parentheses represent the resistance to ofloxacin

Table S7 The distribution of gene mutations associated with amikacin and kanamycin resistance

rrs mutation (codon position)	No. of isolates with resistant phenotype	No. of isolates with susceptible phenotype
a-1401g	5	0
WT	1	118
Total	6	118

Note: WT indicates wild type
